# The law on oncological oblivion in the Italian and European context: How to best uphold the cancer patients’ rights to privacy and self-determination?

**DOI:** 10.1515/med-2025-1222

**Published:** 2025-06-10

**Authors:** Dalila Tripi, Susanna Marinelli, Federica Spadazzi, Francesca Romana Guarnaccia, Simona Zaami, Paola Frati

**Affiliations:** Department of Anatomical, Histological, Forensic and Orthopedic Sciences, Sapienza University, 00161, Rome, Italy; School of Law, Polytechnic University of Marche, Ancona, Italy; Department of Anatomical, Histological, Forensic and Orthopedic Sciences, Sapienza University, Viale Regina Elena 336, 00161, Rome, Italy

**Keywords:** cancer survivors, cancer oblivion, right to privacy, self-determination, ethical/legal implications

## Abstract

**Introduction:**

Italy’s oncological oblivion law codifies the right for cancer survivors to choose not to disclose their medical history under certain conditions, after 10 years from the end of treatment, or 5 years if diagnosed under the age of 21, in order to prevent discrimination in social, work, and professional opportunities.

**Materials and methods:**

This article compares Italy’s law with other European countries’, aiming to analyze its implementation 1-year post-enactment and its medicolegal implications. Official sources and research from platforms like Google Scholar and PubMed were used.

**Results:**

Oncological oblivion was first mentioned in the EU’s General Data Protection Regulation and further emphasized in the European Parliament’s 2020 resolution. Italy’s Law No. 193, enacted on December 7, 2023, aligns with constitutional principles to ensure freedom and equality for cancer survivors.

**Discussion:**

Balancing privacy rights with collective security is crucial, especially in high-risk professions or insurance, where data concealment could raise ethical and legal issues.

**Conclusions:**

In conclusion, while the initiatives implemented thus far are cutting-edge, there is an increasing necessity for more effective protection of the rights to privacy, self-determination, and psychological support to stave off patient discrimination. This is essential to ensure true equality among insured individuals.

## 1 Introduction

### 1.1 What is oncological oblivion

Oncological oblivion is a legal concept referring to the right of a person cured of cancer to avoid disclosing their medical history, in the absence of signs of recurrence, especially in the context of insurance, banking, or employment contracts, after a certain number of years post-recovery. As is well-known, to access insurance policies, loans, adoptions, and sometimes even in the workplace, applicants are required to provide numerous documents and complete various declarations, including health status questionnaires or anamnesis forms. These documents are intended to inform the insurance company, bank, or financial intermediary about the applicant’s past and present health condition, the existence of any diseases, including congenital ones, surgeries undergone, the presence of malformations, and any past traumas or injuries. In this way, the applicant is “obliged” to declare the presence of serious illnesses, whether current or past, which not only compromises their privacy but also their chances of receiving equal treatment compared to others of the same age and gender who have never had such diseases [1,2].

### 1.2 Cancer patients and survivors: The numbers in Italy

In 2024, Italy is expected to have 390,100 new cancer diagnoses: 214,500 in men and 175,600 in women. These numbers are stable compared to the previous 2 years (391,700 in 2022 and 395,900 in 2023). However, mortality is decreasing, thanks to advances in treatments and early diagnosis. In fact, by 2024, approximately 3.7 million people are living after a cancer diagnosis. Regarding childhood cancers or those diagnosed during development, these account for about 2% of the oncological cases observed annually, and in 80% of these cases, patients who had cancer during childhood or adolescence achieve complete remission [3].

Over the last 30 years, as noted in the opinion of the Ethical Committee of the Veronesi Foundation and based on data published in the National Oncology Plan 2023–2027, the development of new diagnostic and treatment pathways has allowed for a constant increase in the population of cancer survivors. It is now estimated that, 5 years after a cancer diagnosis, three out of five individuals are still alive, with even more encouraging data for pediatric patients. In Europe, there are 20 million people still alive after a cancer diagnosis, with 35% of them being part of the so-called “long-term survivors” or “Survivors” group. It is also essential to promote targeted interventions aimed at reducing stigma and encouraging cancer patients to seek support, with the goal of improving their psychosocial adjustment and quality of life. Moreover, integrating mental health services into oncology care and tackling the prejudices associated with psychological support represent crucial steps in enhancing patient outcomes and ensuring comprehensive care [4–9].

## 2 Materials and methods

The aim of this study was to analyze the text of the Italian law on the oncological oblivion, enacted in 2023 and currently being implemented through the adoption of implementing decrees in 2024. The goal was to identify the innovations introduced by the law, highlighting both its positive aspects and the existing critical issues.

The authors first focused on the legislation within the Italian context, grounded in the Constitution, and then compared it to the European framework. This includes recommendations and national legislations that align with the principles set out in the EU Charter of Fundamental Rights [10,11], the EU’s research and innovation program for 2021–2027 (Horizon Europe) [12], and the European mission against cancer within Horizon Europe [13]. These efforts were made in collaboration with other member states to ensure equal rights for all citizens, both sick and healthy.

The analysis was conducted through consultations of the Italian and European Official Gazette, as the official source of current regulations, and by using search engines (Google Scholar; PubMed) with keyword combinations such as “cancer survivors” – “oncological oblivion” – “privacy”; “self-determination” – “ethical/legal implications.”


**Informed consent:** Not applicable for studies not involving humans.

## 3 Results

### 3.1 Italian law – LAW no. 193 of December 7, 2023

As early as 2019, the Italian legal system made progress regarding advance treatment directives and shared care plans, marking a fundamental and essential beginning for every citizen’s right to self-determination, already guaranteed by the Italian Constitution. In 2023, Europe, and subsequently Italy, further expanded the concept of protection for cancer patients by codifying the right to oncological oblivion [14–19].

The Italian law of December 7, 2023, n.193, assuming that cancer is an objectively curable disease, aims to prevent any form of discrimination against former cancer patients by removing obstacles that prevent them from enjoying a range of rights on equal terms, as set out in Article 3, paragraph 2 of the Italian Constitution [20]. As already mentioned, data show that while the number of cancer diagnoses is increasing, survival expectations after a cancer diagnosis are also rising. In 2024, Italy formally introduced the right to oblivion with a law allowing former cancer patients to withhold their health status in banking, financial, investment, and insurance contracts after 10 years from the end of treatment (or 5 years if diagnosed under the age of 21). The law, published in the Official Gazette on December 18, 2023, came into effect on January 2, 2024, aiming to prevent discrimination and protect the rights of those who have been affected by cancer, ensuring equality of treatment. The unified text consists of five articles addressing the subject, objectives, and definitions (Article 1), access to banking, financial, investment, and insurance services (Article 2), amendments to Law No. 184 of May 4, 1983, on adoption (Article 3), access to bankruptcy procedures, employment, and professional training (Article 4), and final and transitional provisions (Article 5).

In more detail, Article 2 defines a 10-year period after the end of active treatment, in the absence of relapses, during which there is no obligation to disclose a general oncological condition (5 years if the condition was diagnosed before the age of 21). Article 2 of the approved text stipulates that for the conclusion or renewal of contracts relating to financial, banking, investment, and insurance services, the request for information about the applicant’s health status concerning previous oncological conditions, whose active treatment has concluded without relapse for more than 10 years, cannot be made. This period is halved if the condition occurred before the applicant turned 21. If health status information has been previously provided, it cannot be used for risk assessment or creditworthiness purposes once the term specified in Article 2 has passed. Therefore, the data must be erased upon submission of a certification by the contractor, confirming the necessary requirements for the application of the law. Violation of these provisions will result in the nullity of individual non-compliant clauses and those connected to them but will not invalidate the entire contract, which remains valid and effective for the rest. The nullity applies only in favor of the consumer and can be raised *ex officio* at any stage of the proceedings.

In the area of adoption (Article 3), the same temporal criteria for the right to oncological oblivion must be applied in relation to the health assessments of applicants, as provided by Law 184/1983.

Regarding access to bankruptcy procedures, employment, and professional training (Article 4), it specifies that if psycho-physical or health status assessments are required within these procedures, it is prohibited to request information about the health status of the applicants concerning oncological conditions they previously had, whose active treatment ended without relapse for more than 10 years from the request date (reduced to 5 years if the condition occurred before the applicant turned 21).

Finally, Article 5 of the law assigns the task of overseeing the application of the right to oncological oblivion to the Data Protection Authority (commonly known as the Privacy Guarantor). The Authority can receive complaints, reports, and grievances from citizens and is endowed with inspection and sanctioning powers. In any case, the violation of the right to oncological oblivion can be challenged before the competent judicial authority (usually the ordinary civil judge, but it could also be the labor judge or the administrative judge for actions taken by a Public Administration) to eliminate prejudice and also provide compensation for damages caused by illegal conduct [21,1].

### 3.2 Legislative decrees issued by the Ministry of Health and the Ministry of Justice in 2024

In order to clearly outline the implementation and application of the law, three legislative decrees were issued in 2024:

- The decree of March 22, 2024 [22], issued by the Ministry of Health, introduced specific tables for individual oncological conditions. These tables establish shortened periods for the onset of the right to oblivion compared to the previously mentioned timeframes, depending on the clinical peculiarities and the likelihood of recovery for each specific pathology (Table 1).
- The decree of July 5, 2024 [23] introduced important legal provisions regarding the submission of requests and the issuance of certificates confirming the “oncological oblivion” status. The application can be submitted to a public or private accredited facility, a specialist doctor employed by the National Health Service (SSN) responsible for the oncological condition for which oblivion is being requested, a general practitioner, or a pediatrician. The certificate, if the requirements set by the applicable regulations and the implementing decree are met, must be issued within 30 days of the application submission, at no cost to the applicant. Regarding personal data management, the oncological oblivion request must be kept for 10 years from the submission date, while the certificate must be retained for 10 years from its receipt. After this period, the holder must proceed with the deletion of the documentation.
- The decree of August 9, 2024 [24], issued by the Ministry of Health in collaboration with the Ministry of Justice, introduced provisions regarding adoption: individuals applying for adoption who are oncological patients, and for whom the terms set by Law 1983, No. 184 [25] (Article 22, paragraph 4) have passed, must provide the health authority conducting the investigation required by the court with the “oncological oblivion” certificate, as outlined in the aforementioned decree. If the deadlines set by Article 22 of Law 1983 are reached after the completion of the investigations by the health authority, the oncological oblivion certificate must be filed with the court where the adoption application was submitted.

**Table 1:** Pathology-specific tables

Patient age	Type of neoplasm	Time of oncological oblivion
Any age	Colorectal neoplasm stage I	1 year from the end of treatment
Age over 21 years	Colorectal neoplasm stages II and III	7 years from the end of treatments
Age >21 years old	Melanoma	6 years from the end of treatments
Any age	Breast cancer stages I and II	1 year from the end of treatments
Age >21 years old	Cervical neoplasms	6 years from the end of treatments
Age >21 years old	Neoplasms of the body of the uterus	5 years from the end of treatments
Any age	Testicular neoplasms	1 year from the end of treatments
Women aged <55 years old	Thyroid neoplasms (excluding anaplastic tumors)	1 year from the end of treatments
Men aged <45 years	Thyroid neoplasms (excluding anaplastic tumors)	1 year from the end of treatments
Age <45 years old	Hodgkin’s Lymphomas	5 years from the end of treatments
Any age	Acute leukemias (lymphoblastic and myeloid)	5 years from the end of treatments

### 3.3 Legislation in Europe: France, Belgium, Luxembourg, Holland, Portugal, Romania

The European Union, through recommendations and harmonized national legislations, aims to establish guidelines that respect the dignity of cured individuals, promoting policies that enhance their autonomy and privacy. This approach is in line with the principles enshrined in the EU Charter of Fundamental Rights [10,11], the EU research and innovation program 2021–2027 (Horizon Europe) [20], and the European Mission Against Cancer under Horizon Europe [21]. The European Parliament Resolution 2020/2267 highlights that “oncological patients should not suffer a ‘double punishment’ in their daily life,” calling for the adoption of a directive against discrimination and the fair and just implementation of financial services directives, such as those related to credit contracts, without discrimination against oncological patients and cancer survivors [26].

France was the first to introduce specific legislation on the oncological oblivion with a 2016 protocol integrated into the Code des Assurances. This law was part of the 5-year cancer plan (2014–2019), developed based on a report by the distinguished French hematologist Professor Jean-Paul Vernant, with the main goal of reducing inequalities and lost opportunities due to cancer. The law stipulated that former cancer patients would not be required to disclose their previous cancer history to insurers or lending agencies after 10 years from the end of their treatment, provided there were no recurrences (or 5 years for those who had cancer before reaching adulthood) [27,28].

Following the French model, Belgium, Luxembourg, the Netherlands, Spain, and Portugal introduced similar regulations to protect former cancer patients. Specifically, in 2022, Belgium, Luxembourg, and the Netherlands enacted legislation to eliminate discrimination in insurance and banking contracts, preventing these companies from requesting information about an individual’s cancer history after a certain period of time post-recovery (10 years from the end of treatment without recurrences or 5 years in cases of cancer diagnosed before adulthood). Spain and Portugal have passed recent laws that include the oncological oblivion as part of broader patient rights protection policies. In 2023, Spain approved regulations guaranteeing the protection of health data and preventing discrimination in financial and employment sectors, while Portugal provides similar guarantees with a focus on insurance and mortgages. Romania is one of the last countries to adopt this legislation, ensuring protections for former cancer patients comparable to those in other European countries [29–31].

## 4 Discussion

The oncological oblivion law, 1 year after its implementation, raises a series of ethical and medical-legal reflections that are particularly relevant in the context of increasing attention to patient rights, related to privacy and the management of health information, with obvious repercussions on the psyche of individuals who suffer from a significant psychological biological damage of medical-legal relevance.

### 4.1 Right to privacy and protection of personal data

The core of oncological oblivion lies in the right to privacy, which is a fundamental principle both ethically and legally. The concept of oncological oblivion is particularly relevant in the context of forensic medicine, as it mainly concerns the management of patients’ rights, privacy, and protection from discrimination related to their oncological medical history. The General Data Protection Regulation (EU) 2016/679 [32] – Article 17 – establishes that data must be deleted when deemed no longer necessary for the original purposes and in the absence of other legitimate reasons to retain it. However, the right to erasure is not absolute. There are exceptions, such as when deleting the data would compromise freedom of expression or if the data must be retained to comply with a legal obligation or for reasons of public interest.

From a medico-legal perspective, failure to protect privacy can generate liability for healthcare institutions or individuals who process data in a non-compliant manner. Furthermore, the absence of an effective oncological right to oblivion can be considered a violation of the patient’s fundamental rights, exposing institutions to legal challenges and compensation claims for moral or material damages.

### 4.2 Self-determination and individual freedom

The principle of self-determination is closely linked to the right to choose which parts of one’s medical history to share with others [33,34]. The right to oncological oblivion allows the patient to avoid being forced to disclose information that may be irrelevant and, at the same time, harmful in the present, especially if the disease has been treated and no longer poses a risk to the patient. In the healthcare field, this principle is particularly important because it implies that each patient should have the freedom to decide which health-related information to share with others.

From a medico-legal perspective, the failure to protect the right to oblivion can constitute a case of indirect liability for those who manage sensitive information, especially if it is used in a discriminatory or improper manner. The lack of a clear regulatory framework or adequate measures to protect the patient could exacerbate the harm, affecting not only their privacy but also their perception of moral and social integrity. Ultimately, protecting the right to oncological oblivion becomes essential to prevent harm from a lack of self-determination, ensuring that the patient has full control over their personal information and guaranteeing that they can live without the burden of a condition that has been overcome, in line with the principles of dignity and equality upheld at the European and international levels.

### 4.3 Potential risks of discrimination

A critical point concerns the discrimination an oncological patient might face if forced to disclose their past condition. For example, insurance companies might apply higher premiums to those who have had cancer, even if cured, due to biases related to perceived risk. This – like the gender discrimination still present in various workplace and insurance contexts [35] – could violate the principle of equality and social justice, as it would create inequalities between those who have had a serious illness and those who never have, even though the former may be considered fully healed. Oncological oblivion, on the one hand, protects the patient from such discrimination, but on the other, it can be seen as a limitation to transparency in professional or insurance contexts. Insurance companies or employers may view the failure to disclose health information as a risk to their business or the safety of colleagues, raising questions about how the right to privacy might conflict with other needs for transparency. The concept of discrimination related to oncological disease is complex and intersects with various social, economic, and psychological issues. The law on oncological oblivion serves as a tool to protect cured patients from potential discrimination, allowing them not to disclose their oncological history unless strictly necessary. However, despite the protective intentions of this law, there are potential risks of discrimination that could arise, especially when health information is concealed or not disclosed transparently.

### 4.4 Psychological implications

Another important aspect concerns the psychological consequences for the patient. The right to oncological oblivion and psychological harm are deeply interconnected, as the failure to protect the former can lead to significant psychological suffering for patients cured of cancer. Living with cancer or the fear of recurrence can be a traumatic experience, and the possibility of not being socially identified as an “oncological patient” can significantly improve quality of life and psychological well-being. For a former oncological patient, constant exposure to their clinical past can serve as a continuous reminder of the period of illness, with psychological effects including:

(a) “Anxiety and stress”: Many cancer survivors experience persistent negative moods, such as cancer-related fears, post-traumatic stress, anxiety, or depression [36]. The obligation to disclose one’s oncological history in areas such as work or insurance can generate insecurity and fear of being discriminated against.
(b) “Stigmatization and social isolation”: Being treated differently due to a past cancer diagnosis can induce feelings of exclusion and shame.
(c) “Loss of personal control”: The inability to choose if and when to share personal information can make the patient feel powerless, exacerbating psychological distress.
(d) “Reactivation of trauma”: Repeated exposure to the clinical past can reopen emotional wounds, preventing the patient from fully embracing their state of recovery. Fear of cancer recurrence is a significantly distressing problem that affects cancer survivors and places the individual at risk for depression, impaired daily functioning, and subsequent reduced quality of life [37,38].

In light of all of such elements characterizing the mental and psychological distress which a large share of cancer patients come to experience, it is essential to frame and put in place new tailored sets of measures which must unfold within the context of mental health-related cancer stigma reduction [39,40]. Such interventions, along with related organic processes and assessment blueprints, need to focus on the causes and effects of the mental health issues and stigma for cancer patients. These factors may greatly vary according to personal traits, types of cancer, social and economic settings, pre-existing or co-existing mental conditions, and vulnerabilities, among other key factors [41,42]. Women who survived gynecological tumors, for instance, may become severely distressed by the prospect or possibility of losing their reproductive capabilities [43], or out of fear of discrimination when trying to access medically assisted reproduction [44,45]. All interventions aimed at providing diagnostic and therapeutic support to meet such challenges need to be cogently assembled by relying on grounded theory studies, in synergy with evidence-based practice more broadly [46]. In that regard, given the set of complexities at hand when providing care to cancer survivors and the wide array of distinctive traits thereof, tools such as the Health Stigma and Discrimination Framework [47], i.e., a framework based on theory, research, and practice, in order to provide well-balanced, innovative and alternative avenues for the conceptualization and response to health-related stigmas (not limited to cancer), deserve to be considered. As for psychological distress or damage from a medico-legal perspective, such notions can be identified as a consequence of a violation of the right to privacy or the failure to apply the right to oncological oblivion. The patient may seek compensation if they can demonstrate that the unauthorized disclosure of their information caused a negative impact on their mental health or compromised their ability to achieve a better outcome than what was achieved. In this sense, psychological damage, as with many other stressful situations [48–50], exacerbated by the traumatic experience related to the neoplastic disease [51,52], is not just an individual matter, but also a major potentially life-changing responsibility for institutions that handle data inadequately.

## 5 Conclusions

The right to oncological oblivion represents a crucial step toward the protection of the rights of individuals cured of cancer, promoting an inclusive and non-discriminatory society. Its implementation in various European countries demonstrates a growing sensitivity to the need to protect the dignity, privacy, and self-determination of those who have overcome a serious illness. However, significant challenges remain, both ethically and legally, related to transparency, fairness, and the balance between individual rights and collective needs, such as security or the collection of epidemiological data.

The protection of patient privacy must be balanced with the obligation of transparency toward institutions and third parties involved, without infringing on the rights of others. Additionally, the lack of knowledge of previous medical information could affect medical, insurance, or employment suitability assessments, raising potential conflicts between the principles of personal autonomy and public safety. The ethical and psychological implications emphasize the importance of an integrated approach that considers not only regulatory needs but also the social and emotional impact of recovery.

To achieve full inclusion and effective protection, it is essential to continue working on shared policies that combine ethical sensitivity, social responsibility, and a global vision of human rights. In the medico-legal field, there is a need to balance the right to privacy with the protection of collective and individual safety, considering that, in specific contexts such as high-risk professions or insurance procedures, the concealment of data could create ethical or legal issues. This process requires interdisciplinary collaboration among legislators, healthcare professionals, ethics, and legal experts, to ensure that the right to oncological oblivion not only protects, but becomes a true tool of emancipation for those who have faced and overcome cancer, strengthening the values of equity and justice in all areas of life.
